# Phosphorylation of islet-1 serine 269 by CDK1 increases its transcriptional activity and promotes cell proliferation in gastric cancer

**DOI:** 10.1186/s10020-021-00302-6

**Published:** 2021-05-07

**Authors:** Qiong Shi, Xiaomei Ni, Ming Lei, Quansong Xia, Yan Dong, Qiao Zhang, Weiping Wang

**Affiliations:** 1 Clinical Laboratory, The Third Affiliated Hospital, Kunming Medical University & Yunnan Cancer Center, Yunnan, Kunming P. R. China; 2 Pathology Department, The Third Affiliated Hospital, Kunming Medical University & Yunnan Cancer Center, Yunnan, Kunming P. R. China; 3 Department of Biochemistry and Molecular Biology, School of Basic Medical Sciences, Kunming Medical University, Yunnan, Kunming P. R. China; 4 Department of Biochemistry and Molecular Biology, School of Basic Medical Sciences, Beijing Key Laboratory of Protein Posttranslational Modifications and Cell Function, Key Laboratory of Molecular Cardiovascular Sciences, Ministry of Education of China, Peking University, Beijing, P. R. China

**Keywords:** ISL1, Phosphorylation, CDK1, Transcription activity, Gastric cancer

## Abstract

**Background:**

Despite recent advances in diagnostic and therapeutic approaches for gastric cancer (GC), the survival of patients with advanced GC remains very low. Islet-1 (ISL1) is a LIM-homeodomain transcription factor, which is upregulated and promotes cell proliferation in GC. The exact mechanism by which ISL1 influences GC development is unclear.

**Methods:**

Co-immunoprecipitation (co-IP) and glutathione S-transferase (GST)-pulldown assays were employed to evaluate the interaction of ISL1 with CDK1. Western blot and immunohistochemistry analyses were performed to evaluate the ability of CDK1 to phosphorylate ISL1 at Ser 269 in GC cell and tissue specimens. Chromatin immunoprecipitation (ChIP), ChIP re-IP, luciferase reporter, and CCK-8 assays were combined with flow cytometry cell cycle analysis to detect the transactivation potency of ISL1-S269-p and its ability to promote cell proliferation. The self-stability and interaction with CDK1 of ISL1-S269-p were also determined.

**Results:**

ISL1 is phosphorylated by CDK1 at serine 269 (S269) in vivo. Phosphorylation of ISL1 by CDK1 on serine 269 strengthened its binding on the cyclin B1 and cyclin B2 promoters and increased its transcriptional activity in GC. Furthermore, CDK1-dependent phosphorylation of ISL1 correlated positively with ISL1 protein self-stability in NIH3T3 cells.

**Conclusions:**

ISL1-S269-p increased ISL1 transcriptional activity and self-stability while binding to the cyclinB1 and cyclinB2 promoters promotes cell proliferation. ISL1-S269-p is therefore crucial for tumorigenesis and potentially a direct therapeutic target for GC.

**Supplementary Information:**

The online version contains supplementary material available at 10.1186/s10020-021-00302-6.

## Introduction

Gastric cancer (GC) is the fifth most common malignancy and the third leading cause of cancer-related deaths(Ferlay et al. 2015). China is among the countries with the highest incidence of GC, accounting for over 40% of all new cases of GC worldwide(Bu and Ji 2013). Despite recent advances in diagnostic modalities and the development of molecular-targeted drugs, the survival rates of patients with advanced GC remains very low(Waddell et al. 2013). Understanding the precise molecular mechanisms underlying GC progression is therefore crucial for the development of effective therapeutic strategies against GC.

ISL1 or human insulin gene enhancer binding protein 1 (islet-1) is a LIM homeodomain transcription factor encoded by the ISL1 gene(Karlsson et al. 1990), and functions as a transcription factor that regulates the expression of genes involved in heart, motor neuron, and pancreas development and foregut morphogenesis(Bu et al. 2009; Guo et al. 2011; Kim et al. 2019; Shi et al. 2009; Zhang et al. 2018). ISL1 is a marker for pancreatic neuroendocrine tumors and their metastases(Dong et al. 1991; Wang et al. 2014). We previously reported that ISL1 is highly expressed in GC and is correlated with advanced tumor-nodes-metastasis (TNM) stage, lymph node metastasis, and poorer overall survival(Guo et al. 2015). Further, ISL1 could activate cyclinB1, cyclinB2, and c-myc expression in GC cells by directly binding to conserved binding sites on their promoter or enhancer, and thereby promote GC cell proliferation(Shi et al. 2016). However, the exact mechanisms by which ISL1 modulates GC development remain unknown. Understanding the roles of this protein in GC development and the ways in which it could be modulated could be of great research and clinical interest.

Post-translational phosphorylation plays critical roles in the assembly of signaling and repair proteins in several biological progresses (Sharma et al. 2014). Although several phosphorylation sites such as T182 (Zahedi et al. 2008) and S221(Mertins et al. 2013; Sharma et al. 2014) have been identified in ISL1 proteins, but the molecular mechanisms that govern their physiological effects remain unclear. Further, ISL1 overexpression or knockdown leads to increased or decreased GC cell proliferation, respectively, and can influence cell cycle transition (Guo et al. 2019; Shi et al. 2016). CDK1 is a major cell cycle transition kinase (McDonald et al. 2020); we therefore investigated whether ISL1 is regulated by CDK1.

In this study, we aimed to elucidate the molecular interactions of endogenous ISL1 with CDK1 in GC cell lines. Our results showed that CDK1 phosphorylated ISL1 Ser-269, which promoted its transcriptional activity and increased protein self-stability. The S^269−^ISL1-CDK1 complex increased the proliferation in GC cells. Collectively, our results provide the first evidence that CDK1 phosphorylates ISL1 Ser-269, and provides insights into the role of ISL1 in GC.

## Methods and materials

### Cell lines and culture

The GC cell line BGC823 (ICLC: HTL98007), was purchased from Instituto Nazionale per la Ricerca sul Cancro (ICLC, Genova, Italy). MGC803, MKN28, and an immortalized human gastric epithelial cell line, GES1, were gifts from Profs. Shou CC and Ke Y (Peking University Cancer Hospital). Cells were cultured in Dulbecco’s modified Eagle’s medium (DMEM) supplemented with 10% fetal bovine serum (FBS), 80 U/ml penicillin, and 100 mg/ml streptomycin at 37 °C under 5% CO_2_.

### Immunoprecipitation and western blot analysis

Cell lysates were prepared using radioimmunoprecipitation assay (RIPA) lysis buffer (P0013E; Beyotime, Shanghai, China) supplemented with protease inhibitor cocktail (469,313,200; Roche, Basle, Switzerland). Immunoprecipitation and western blot analysis were carried out as described previously (Liu et al. 2009), with ISL1 for Co-IP (H00003670-M05, Abnova, Taipei, China) and ISL1 for western blotting (ab109517, Abcam). Monoclonal anti-CDK1 (#9116), GAPDH (#2118), rabbit polyclonal anti-CDK1-T161P (#9114), and mouse monoclonal anti-cyclin B1 (#4135) were purchased from Cell Signaling Technology (Danvers, MA, USA). Mouse monoclonal anti-phosphoserine (05-1000) was obtained from Merck Millipore (Millipore Corp, Billerica, MA, USA). Rabbit polyclonal antibody against the phosphorylation site of ISL1 at Ser 269 (ISL1-S269) (1:1,000) was produced with the synthetic phosphopeptide MTGTPMVAA[Sp]PERHDGGLQ. (Absin Bioscience Inc., Shanghai, China)

### Glutathione S-transferase (GST) pull‐down assay

GST fused with full-length ISL-1 or various truncated ISL-1 proteins were prepared as described previously (Zhang et al. 2009). Biotin-labeled wild-type CDK1 was synthesized using the TNT Quick Coupled Transcription/Translation System (L5020, Promega, Madison, WI, USA) (Wang et al. 2016).

## Immunohistochemistry (IHC)

All human GC tissue specimens (35 poorly differentiated adenocarcinoma, 22 moderately differentiated adenocarcinoma, 3 well-differentiated adenocarcinoma) were obtained from the Pathology Department, School of Clinical Oncology, Kunming Medical University (China). IHC was performed on 5-mm-thick serial sections of formaldehyde-fixed, paraffin-embedded tissue blocks derived from GC with paired adjacent tissue. The sections were incubated overnight at 4 °C with an antibody against ISL1 phosphorylated at Ser 269 (ISL1-S269-p)(1:150) produced using the synthetic phosphopeptide MTGTPMVAA[Sp]PERHDGGLQ (Absin Bioscience Inc, Shanghai, China). The extent of staining was defined as follows: 0%: -, 1–24%: +, 25–49%: ++, 50–74%: +++, and 75–100%: ++++.

## Chromatin immunoprecipitation (ChIP) and ChIP re-IP assays

ChIP and ChIP re-IP experiments were performed in MKN28 and MGC803 cells as previously described(Zhang et al. 2007). After elution, real-time PCR or conventional PCR were performed to amplify the DNA fragment containing the *CCNB1* or *CCNB2* promoters, covering the ISL1/CDK1 binding sites, with the primers listed in Table 1.

**Table 1:** Primer sequences

Gene	Primer sequences (5ʹ–3ʹ)
*CCNB1*	Sense: CCGCTTCGGACTGCGAACTAA
	Antisense: AGAGCAGGCAGCAGCTAAGAAGG
*CCNB2*	Sense: GCGGTATTTGAATCCTGGAACAAG
	Antisense: CGGACTGAAAAGGGAGGACACT

### Plasmid transfection and RNA interference

Cells were transfected with plasmids or synthesized siRNA for gene overexpression or knockdown, respectively. The plasmid constructs pcDNA3.1-ISL1, control pcDNA3.1, pLL3.7-ISL1-siRNA (si-ISL1), and control pLL3.7-Non-silencer have been previously described.(Guo et al. 2011). The pcDNA3.1-ISL1-S269A plasmid was constructed by TransGen Biotech (Beijing, China). RNAi duplexes for CDK1 (si-CDK1) and control siRNA (non-silencing, Non) were obtained from GeneChem (Shanghai, China; Table 2) DNA plasmids encoding CDK1 (Addgene plasmid #1886) and CDK1-DN (Addgene plasmid #1887) were obtained from Addgene. Site-directed mutagenesis was commercially performed by GeneChem (Beijing, China), and Ser 269 in ISL1 was replaced with alanine. Transfection of the plasmids and siRNA was carried out using LipofectamineTM2000 (Invitrogen, Carlsbad, CA, USA) according to the manufacturer’s protocols. After 48 h of transfection, cells were harvested. All transfections were performed at least three times.

**Table 2:** RNAi duplex sequences

Gene	RNAi duplex Sequences (5ʹ–3ʹ)
*CDK1*-1	Sense: CCAUGGAUCUGAAGAAAUA dTdT
	Antisense: dTdT GGUACCUAGACUUCUUUAU
*CDK1*-2	Sense: GCCAGAAGUGGAAUCUUUA dTdT
	Antisense: dTdT CGGUCUUCACCUUAGAAAU
*CDK1*-3	Sense: UCGGGAAAUUUCUCUAUUA dTdT
	Antisense: dTdT AGCCCUUUAAAGAGAUAAU
	Antisense: ACGUGACACGUUCGGAGAAT

### Luciferase assay

Plasmid transfection and luciferase activity detection were performed as described previously (Liu et al. 2011). The cyclinB1-luc and cyclinB2-luc reporters were constructed by TransGen Biotech (Beijing, China).

### Cell proliferation assays

The cell proliferation was assayed using CCK-8 as described previously (Guo et al. 2011).

### Flow cytometry cell cycle analysis

For cell cycle analysis, cells were fixed in ice-cold 70% ethanol and incubated at 4 °C overnight. Fixed cells were washed with phosphate-buffered saline (PBS) and treated with 100 µg/ml RNase A at 37 °C for 20 min. After staining with propidium iodide (50 µg/ml), the cells were subjected to fluorescence-activated cell sorting (FACS) on a FACScan instrument (Becton Dickinson, Franklin Lakes, NJ, USA). Cell debris and fixation artifacts were gated out and cell populations at the G_0_/G_1_, S, and G_2_/M phases were quantified using the ModiFit LT v2.0 software.

### Statistical analysis

Data are expressed as the mean ± standard deviation (SD). All statistical analyses were conducted using SPSS (version 20; SPSS, Chicago, Illinois). *χ*^2^ tests and rank sum tests were used to evaluate the relationship between the ISL1-S629-p immune reaction and the clinicopathological features of the patients. Kaplan-Meier survival curves was generated to evaluate the effect of ISL1-S629-p on patient survival. For all statistical tests, the significance of differences between groups was determined using one-way analysis of variance (ANOVA). Differences were considered statistically significant at *p* < 0.05.

## Results

### ISL1 interacts with CDK1 in vivo and in vitro

**Fig. 1:**
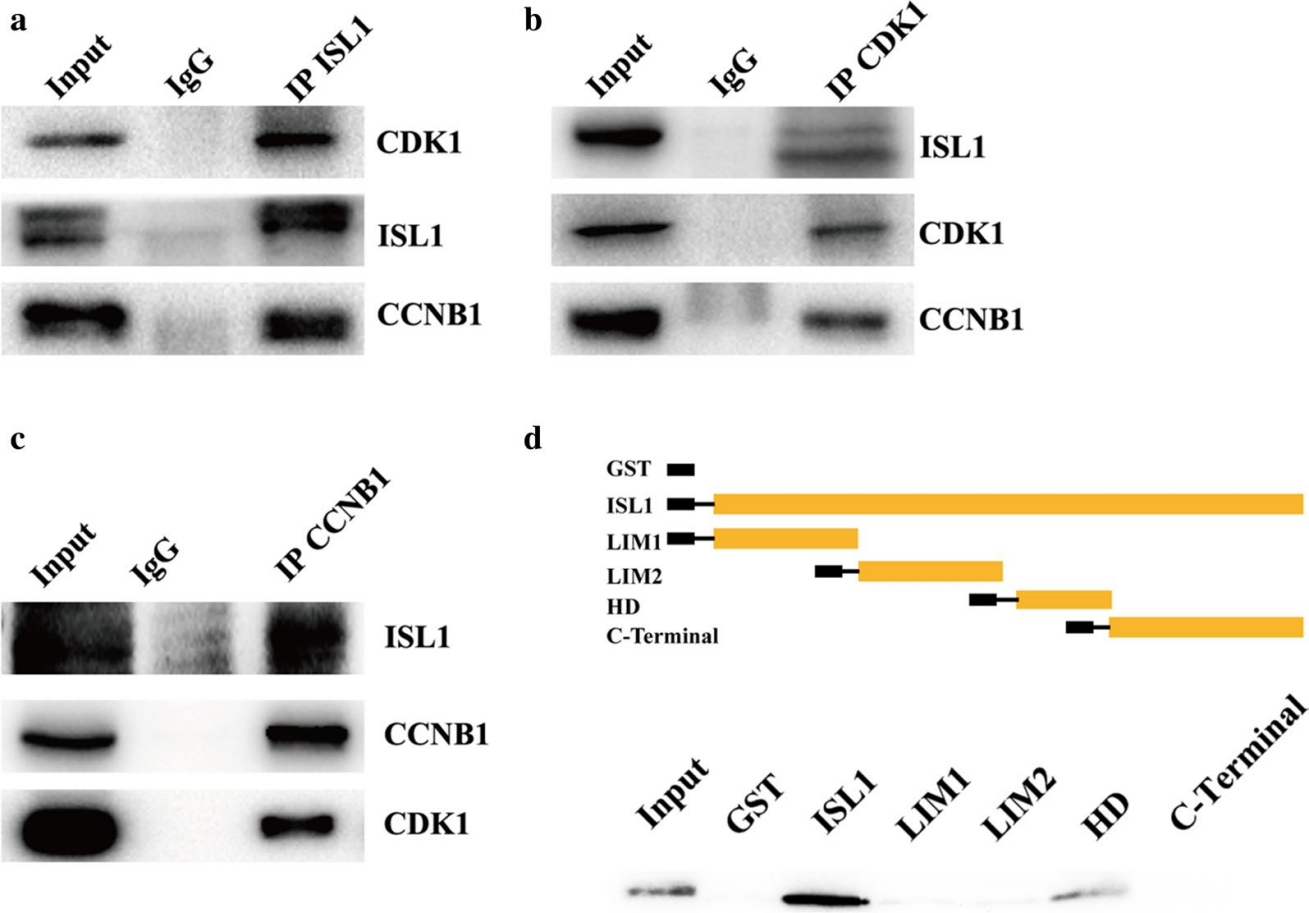
ISL1 interacts with CDK1. **a–c** Co-IP assays were performed to measure the ISL1/CDK1/cyclinB1 complex in MGC803 cells. Lysate was immunoprecipitated with antibodies against ISL1, CDK1, or cyclinB1 and analyzed by immunoblotting. IgG: immunoglobulin G. **d** Full-length or truncated ISL1 was used to construct GST-fusion proteins (Top) for pull-down assays with CDK1 protein (Bottom)

In our previous study, we used mass spectrometry (Lu et al. 2015) to show that ISL1 interacts with CDK1 in MGC803 cell lines (high levels of ISL1 and higher proliferation rates) (Shi et al. 2016). Here, we determined whether ISL1 physically interacts with CDK1/cyclin B1 by using reciprocal co-immunoprecipitation. Our results indicated that these two molecules interact in vivo (Fig. 1a–c). We also used GST linked to ISL1 segments or GST alone to validate the interaction of CDK1 and ISL1 by using a GST pull-down assay; our data suggested that ISL1 interacts directly with CDK1 (Fig. 1d).

### CDK1 mediates ISL1 phosphorylation at serine 269 in GC

**Fig. 2:**
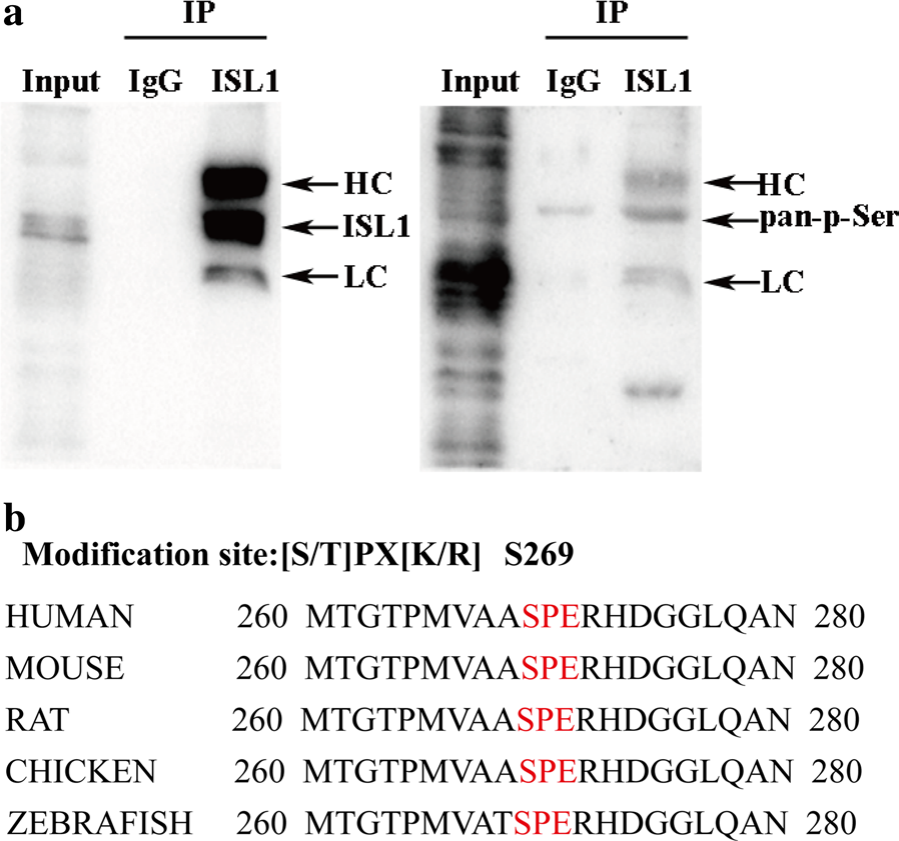
ISL1 is serine-phosphorylated. **a** MGC803 cells lysates were immunoprecipitated (IP) using anti‑ISL1 and analyzed by immunoblotting using antibody against phosphorylated serine and ISL1 (HC: heavy chain, LC: light chain). **b** Alignments of the cluster of ISL1; S269 fit the CDK1 phosphorylation consensus, and (S/T)PX(R/K) sequences (red shade) are shown

To determine whether ISL1 is phosphorylated, we performed co-immunoprecipitation using antibodies against phosphorylated serine and ISL1, and observed phosphorylation serine in ISL1 (Fig. 2a). We then sought to determine which residue in ISL1 could be phosphorylated by CDK1. CDKs often recognize and phosphorylate the serine/threonine-proline (S/T)PX(R/K) motif (Zhang et al. 2008). There is only one such motif (S269/P270) present in ISL1 (Fig. 2b), which matched with CDK phosphorylation consensus sites.

**Fig. 3:**
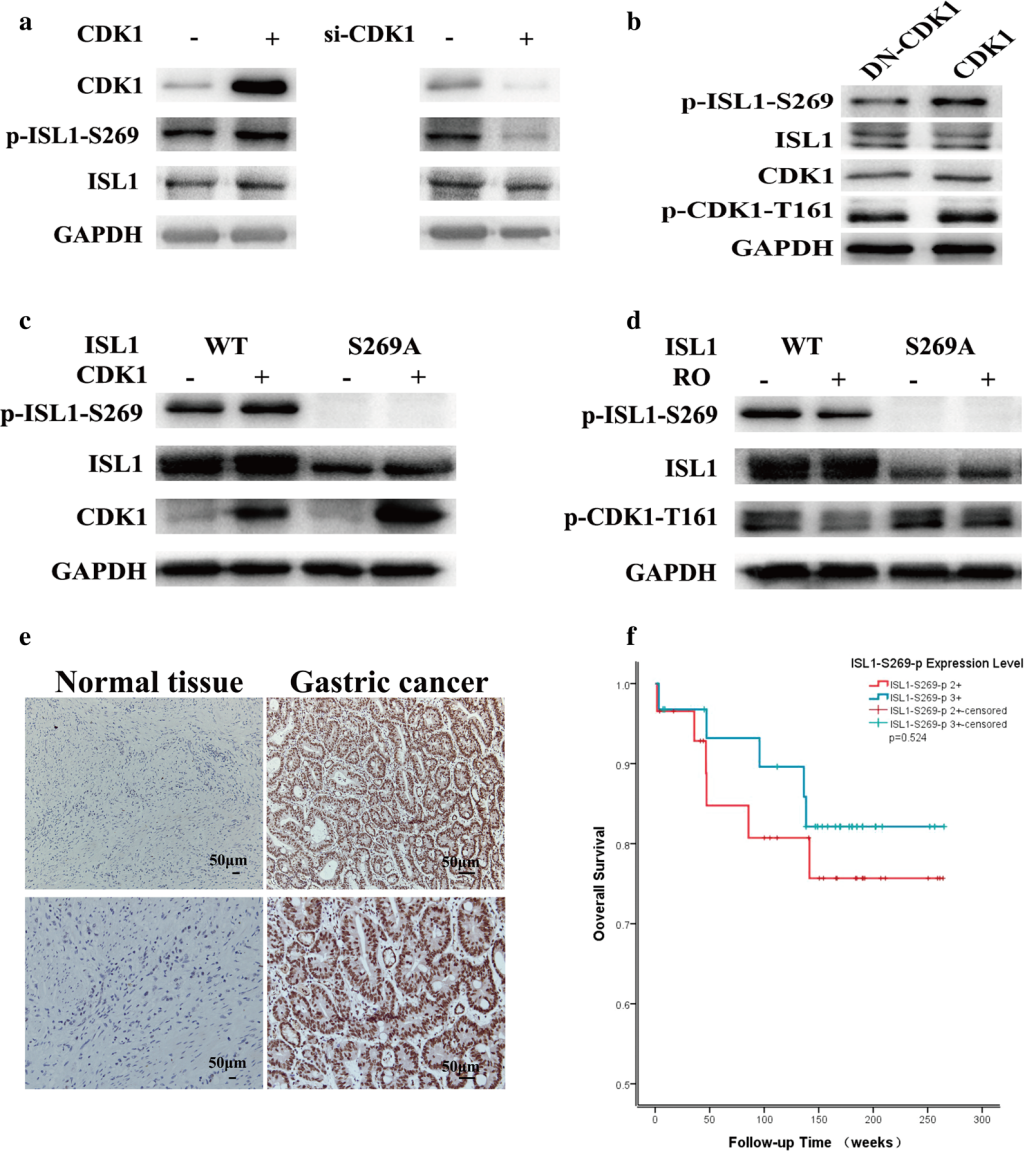
CDK1 mediates ISL1 phosphorylation at serine 269. **a** MGC803 cells transfected with plasmid encoding CDK1 or control vector (left), negative control or CDK1 si-RNA (right). Lysate was analyzed by immunoblotting with antibodies against the indicated proteins. **b** MGC803 cells were transfected with plasmids encoding dominant‑negative mutant CDK1 (DN‑CDK1) or CDK1, and lysate was analyzed by immunoblotting with antibodies against the indicated proteins. **c** MKN28 cells expressing wild-type ISL1 or ISL1-S269A were transfected with plasmid encoding CDK1 or control vector. Cell lysates were immunoblotted with antibodies against the indicated proteins. **d** MKN28 cells were transfected with plasmids encoding wild-type ISL1 or ISL1-S269A and treated with CDK1 inhibitor RO-3306 or DMSO. Lysates were analyzed by immunoblotting with antibodies against the indicated proteins. GAPDH served as the internal control. **e** Representative IHC staining of ISL1-S269-p expression in GC (right, n = 60) and paired adjacent tissues (left, n = 60). **f** Relationship between expression of ISL1-S629-p and overall survival in gastric cancer

Next, we confirmed that CDK1 phosphorylates ISL1 at Ser 269 in vivo. We used a phospho-ISL1-Ser 269 antibody to immunoblot lysates from MGC803 cells transfected with plasmids encoding CDK1 or CDK1 siRNA. The endogenous level of phosphorylated ISL1 at Ser 269 increased when CDK1 was overexpressed, and *CDK1* knockdown significantly reduced Ser 269 phosphorylation (Fig. 3a). Similar changes were also observed in the human gastric epithelial cell line GES1 (Additional file 2: Fig. S2). Furthermore, expression of dominant-negative mutant CDK1 (DN-CDK1) also decreased Ser 269 phosphorylation (Fig. 3b). ISL1 was visualized as two bands in the western blots of some samples. These two bands may represent the alternatively spliced variants, ISL1α and ISL1 β, which have been reported previously (Ando et al. 2003). These results indicate that Ser 269 is phosphorylated by CDK1 in this cell line.

We replaced the Ser 269 residue with alanine (ISL1S269A), and transfected MKN28 cell lines (which have low levels of ISL1 and proliferate slowly) with wild-type or S269A-mutant ISL1. Phosphorylation of ISL1 was examined by immunoblotting with phospho-specific antibody against phospho-S269. Forced expression of CDK1 increased S269 phosphorylation of wild-type ISL1 but not of S269A mutant (Fig. 3c, lane 2 versus lane 4). We also compared the phosphorylation levels of wild-type ISL1 and ISL1 S269A in MKN28 cells transfected with either wild-type (WT) or S269A-mutated ISL1 (S269A), and cultured without or with RO-3306 (10 µM, 20 h, Additional file 1: Fig. S1), a pan-CDK1 inhibitor (Vassilev et al. 2006). Decreased phosphorylation of Ser 269 was observed in cells expressing WT ISL1 cultured with RO-3306 (Fig. 3d, lane 2), with total ISL1 levels remaining constant. Our results indicated that CDK1 increased phosphorylation of Ser 269, and S269A mutation abolished CDK1-mediated phosphorylation of ISL1. In this study, we confirmed the relationship between ISL1-S629-p expression and the overall survival time of patients with GC (35 poorly differentiated adenocarcinoma, 22 moderately differentiated adenocarcinoma, 3 well-differentiated adenocarcinoma) (Fig. 3e). The five-year survival rates of patients with ISL1-S629-p + + and ISL1-629-p+++ were 76 and 82%, respectively. There was no significant correlation between the expression of ISL1-S629-p and overall survival (*p* = 0.524) (Fig. 3f). We also analyzed the relationship between expression of ISL1-S629-p and clinic pathological parameters of GC. Expression of ISL1-S629-p was not significantly correlated with sex, age, Tumor Node Metastasis (TNM) stages, and distant metastasis, but correlated negatively with the degree of invasion (*p* = 0.021) and differentiation of GC (*p* = 0.010) (Table 3).

**Table 3:** Relationship between expression of ISL1-S629-p and clinic pathological parameters of gastric cancer

Characteristic	Expression Level of ISL1-S629-p		p-value
	++(%)	+++(%)	
	(n = 29)	(n = 31)	
Age			
≤ 60	16 (55.2%)	10 (32.3%)	0.073
> 60	13 (44.8%)	21 (67.7%)	
Gender			
Male	19 (65.5%)	20 (64.5%)	0.935
Female	10 (34.5%)	11 (35.5%)	
Depth of invasion			
T1–2	12 (41.4%)	22 (71.0%)	0.021
T3–4	17 (58.6%)	9 (29.0%)	
Lymph node metastasis			
N0	10 (34.5%)	15 (48.4%)	0.275
N1–3	19 (65.5%)	16 (51.6%)	
Distant metastasis			
M0	25 (86.2%)	28 (90.3%)	0.702
M1	4 (13.8%)	3 (9.7%)	
TNM stage			
I	7 (24.1%)	14 (45.2%)	0.309
II	10 (34.5%)	7 (22.6%)	
III	9 (31.0%)	6 (19.4%)	
IV	3 (10.3%)	4 (12.9%)	
Histological grade*			
G1	1 (3.4%)	2 (6.5%)	0.010
G2	6 (20.7%)	16 (51.6%)	
G3	22 (75.9%)	13 (41.9%)	

### CDK1 phosphorylation of ISL1 promotes ISL1 binding on cyclinB1 and cyclinB2 promoters

**Fig. 4:**
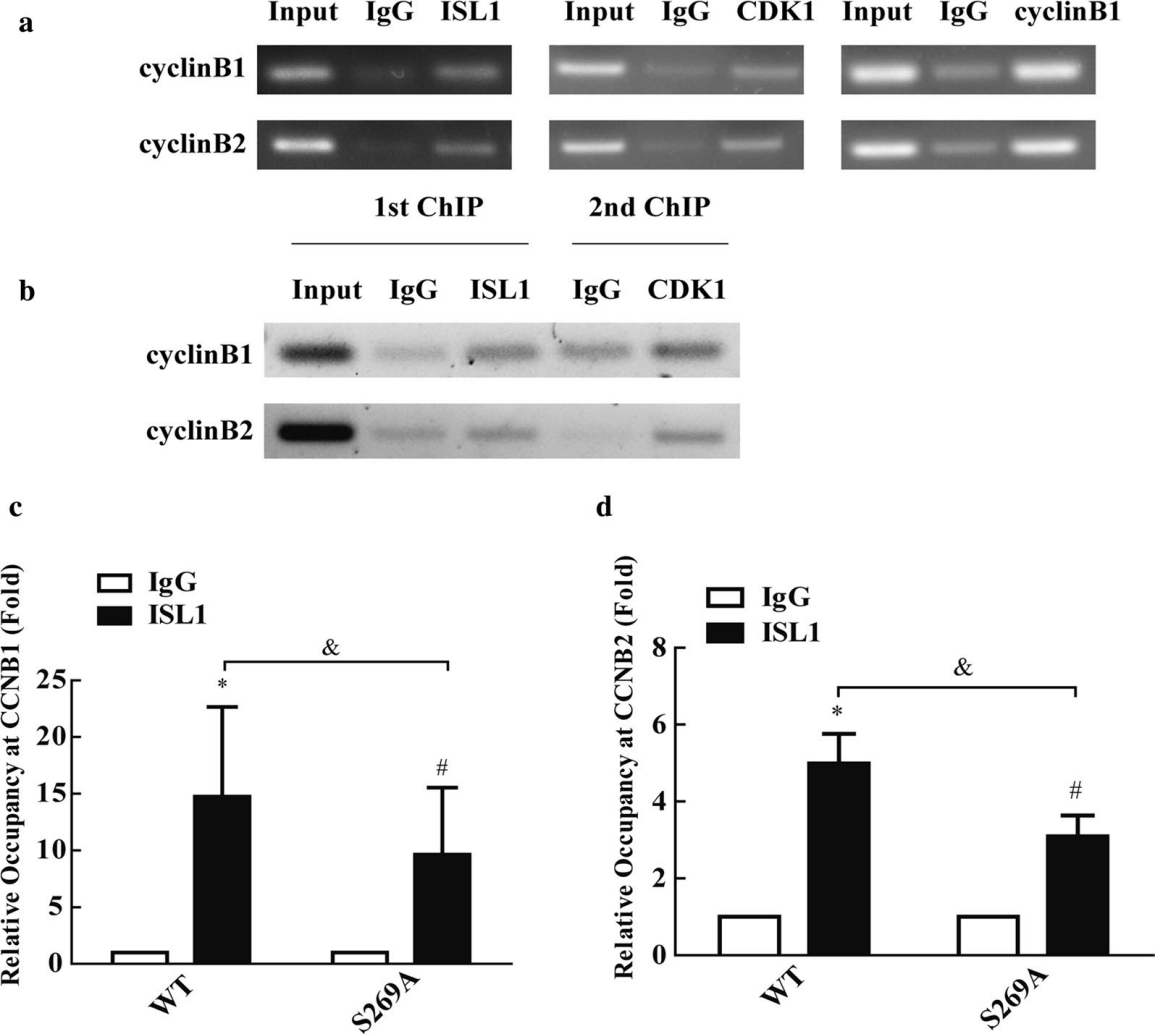
Phosphorylated ISL1 showed improved localization on cyclinB1 and cyclinB2 promoters. **a** ChIP assay analysis and PCR gel electrophoresis analysis of ISL-1 recruitment to the cyclinB1 and cyclinB2 promoters in MGC803 cells. **b** ChIP-re-IP assay was performed with anti-ISL-1 or rabbit IgG antibodies and then with anti-CDK1 or IgG antibodies for immunoprecipitation using chromatin harvested from MGC803 cells. **c**,** d** Quantitative chromatin immunoprecipitation analysis of cyclinB1 and cyclinB2 promoters in MKN28 cell line transfected with control vector or expressing wild-type ISL1 or ISL1-S269A plasmids. Data are expressed as mean ± s. d. from three individual experiments. (*p < 0.05, WT vs. control, ^#^*p* < 0.05, S269A vs. control, ^$^*p* < 0.05, WT vs. S269A)

ISL1 functions primarily as a transcription factor. Our next aim was therefore to determine whether the ISL1-CDK1/cyclin B1 complex binds to the promoters of the target genes *CCNB*1 and *CCNB*2 in MGC803 cells, by performing a ChIP assay. We found that ISL1-CDK1/cyclin B1 formed a transcriptional activation complex with the *CCNB*1 and *CCNB*2 promoters (Fig. 4a, b). Next, we determined whether CDK1-mediated ISL1 phosphorylation affects its transcriptional function. We analyzed ISL1 levels on the promoters of *CCNB*1 and *CCNB*2. Quantitative chromatin immunoprecipitation (qChIP) assay results showed lower levels of S269A-mutant ISL1 (S269A) that of wild-type (WT) on the *CCNB*1 and *CCNB*2 promoters (Fig. 4c, d). These results suggest that phosphorylation of ISL1 at Ser 269 by CDK1 strengthens ISL1 binding on the *CCNB*1and *CCNB*2 promoters.

### Phosphorylation of ISL1 Ser 269 increases transactivation potency and promotes cell proliferation

**Fig. 5:**
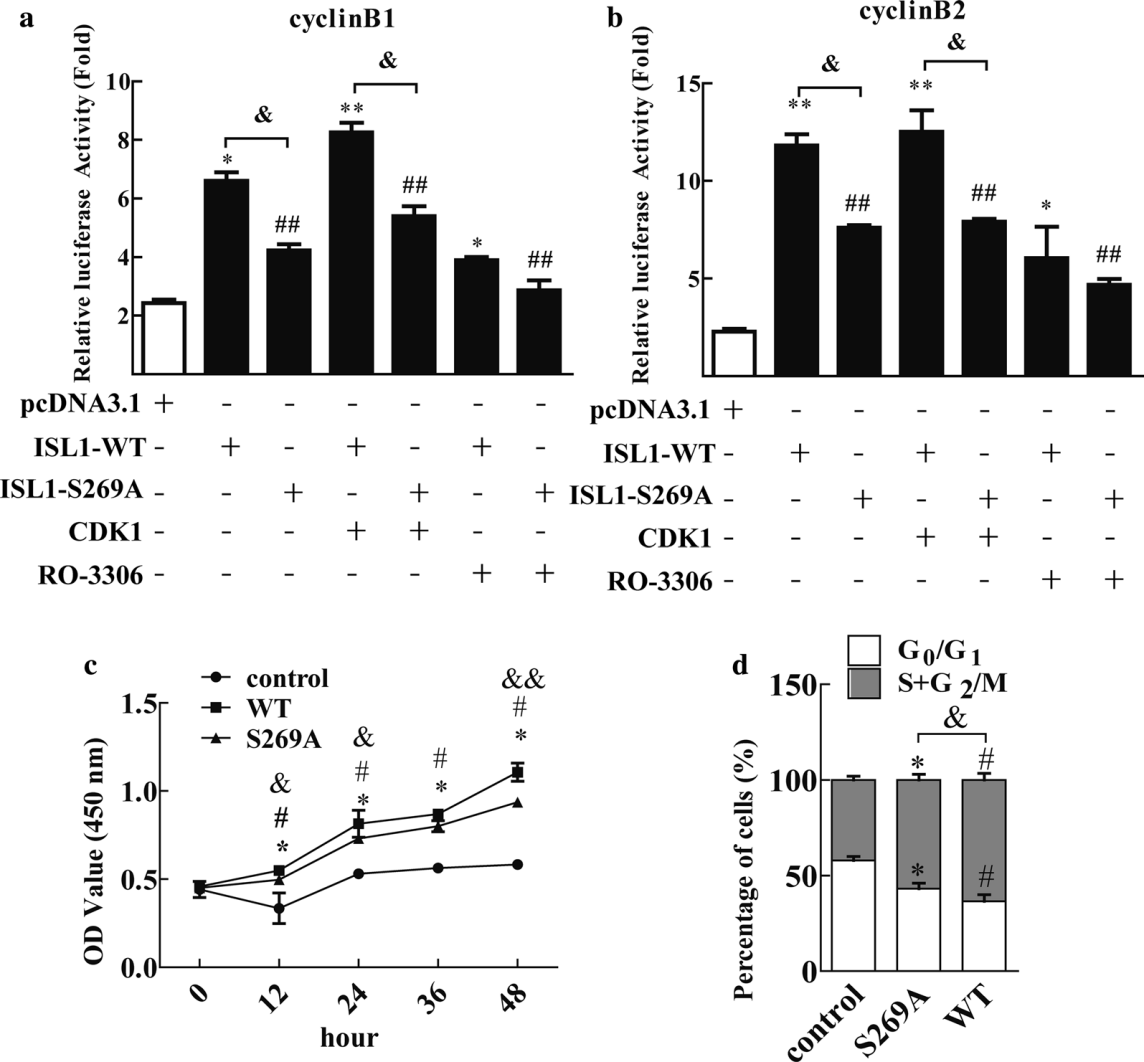
Phosphorylation Ser 269 of ISL1 increases transactivation potency. **a**,** b** Effect of CDK1 activity on the transcriptional activity of wild-type ISL1 or S269A ISL1. Transcriptional activity of wild-type ISL1 and S269A ISL1 on cyclinB1-luc and cyclinB2-luc determined by luciferase reporter assay in MKN28 cells. **c** Cell proliferation was studied by CCK-8 analysis of MKN28 cells transfected with plasmids encoding control vector and wild-type ISL1 or ISL1-S269A. **d** Cell cycle distributions were analyzed by flow cytometry in MKN28 transfected with plasmids encoding control vector and wild-type ISL1 or ISL1-S269A. The data represent 3 independent experiments, each performed in triplicate. Each bar represents mean ± SD. (*p < 0.05, WT vs. control, ^#^*p* < 0.05, S269A vs. control, ^$^*p* < 0.05, WT vs. S269A)

We assayed the transactivation potency of wild-type (WT) or S269A-mutant ISL1 (S269A) by expressing each of these proteins in MKN28 cells along with plasmids encoding CDK1 or CDK1 inhibitor RO-3306. As shown in Fig. 5a, b, constitutive expression of either WT or CDK1/WT (Fig. 5a, b; second column versus fourth column) increased transcriptional activity of ISL1 in MKN28 cells. Similarly, S269A or WT/RO-3306 (Fig. 5a, b; third column versus sixth column) decreased the transcriptional activity of ISL1. Importantly, the enhancement of ISL1 by CDK1 was abrogated by the S269A mutation (Fig. 5a, b; fifth column versus seventh column). ISL1 enhances cell proliferation and cell cycle stimulation. We therefore investigated the effect of the S269A mutation on ISL1-mediated biological functions. By using stable transfects expressing wild-type ISL1 and ISL1 S269A in MKN28 cells, we observed significant differences in cell proliferation and cell cycle stimulation. Wild-type ISL1 and ISL1 S269A cells grew faster than did the control cells, and wild-type ISL1 cells grew faster than did ISL1 S269A cells at 48 h after transfection (Fig. 5c). Moreover, MKN28 cell cycle profiles showed that ISL1 S269A overexpression remarkably decreased the cell population in the G_1_ phase (from 57.94% ± 1.65 to 43.15% ± 2.92, *p* < 0.05) and increased the cell population in the S and G_2_/M phases (from 42.05% ± 1.65 to 56.84% ± 2.92, *p* < 0.05). This effect was significantly more enhanced by transfection with wild-type ISL1 (G_1_ phase from 57.94% ± 1.65 to 36.49% ± 3.48, S and G_2_/M phases from 42.05% ± 1.65 to 63.50% ± 3.48, *p* < 0.05; Fig. 5d). Collectively, our results demonstrated that CDK1-induced phosphorylation of ISL1 Ser 269 is a critical positive regulator of cell proliferation and cell cycle transition.

### ISL1 phosphorylation increases self-stability but does not increase interaction with CDK1

**Fig. 6:**
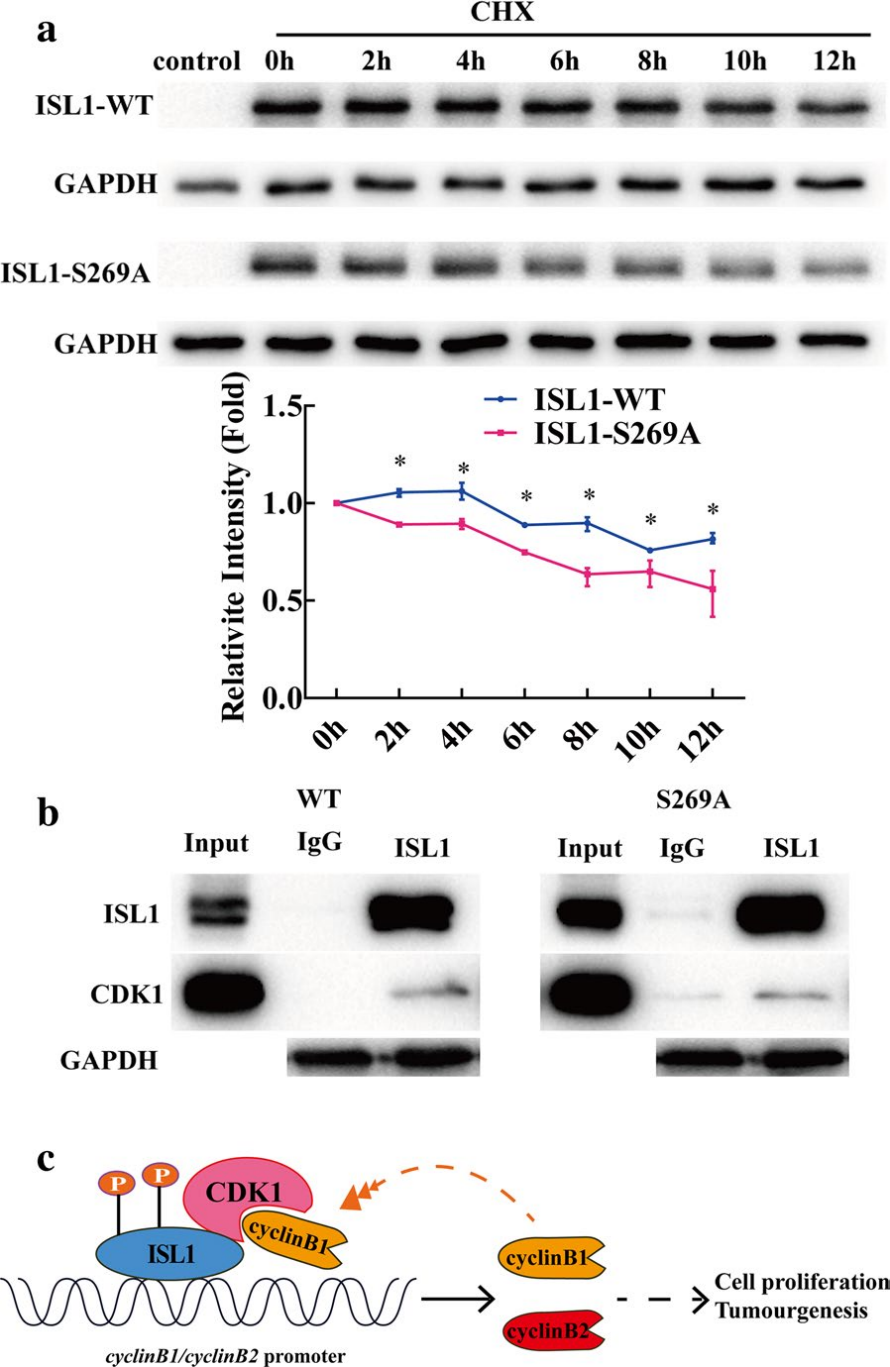
Phosphorylation of ISL1 increases self-stability but does not increase its interaction with CDK1. **a** NIH3T3 cells were transfected with wild-type ISL1 or ISL1-S269A plasmids. After 48 h, cells were treated with CHX (100 µg/ml) for 12 h. Whole cell extracts were harvested for western blotting. **b** Co-IP assays were performed in NIH3T3 cells to detect the interaction between ISL1 (wild-type or mutants) and CDK1. GAPDH served as a loading and negative control. **c** Schematic representation of positive feedback regulation by the ISL1-CDK1/cyclin B1/2 complex in GC. Full arrow: confirmed; broken arrow: to be verified

To assess whether ISL1 phosphorylation mediates self-stability, NIH3T3 cells were transfected with wild-type ISL1 or ISL1 S269A for 48 h and treated with cycloheximide (CHX) to block protein synthesis for various lengths of time. Total cell lysates were prepared and immunoblotted. ISL1 S269A protein levels decreased within 6 h, and further reduced 10 h after CHX treatment, while wild-type ISL1 protein levels remained unchanged within 8 h after treatment with CHX (Fig. 6a). We then studied the effect of CDK1 modification on ISL1-CDK1 combination. Nuclear extracts prepared from NIH3T3 cells transfected with CDK1 and either ISL1-WT, or ISL1-S269A for 48 h were subjected to Co-IP assay. We did not observe significant differences in ISL1-CDK1 binding (Fig. 6b). These data suggest that ISL1 phosphorylation increases self-stability but does not increase its interaction with CDK1.

## Discussion

The expression and DNA methylation of ISL1 in carcinoma has been studied in many tumors (Dong et al. 1991; Erlenbach-Wunsch et al. 2014; Graham et al. 2013; Kim et al. 2013; Kitchen et al. 2015; Li et al. 2018; Wang et al. 2014; Zhang et al. 2014). We demonstrated previously that ISL1 expression may serve as a specific marker for GC treatment and prognosis (Guo et al. 2015). Further, ISL1 enhanced tumor growth and targeted the downstream genes *CCNB*1, *CCNB*2, and *C-myc* in GC (Shi et al. 2016). ISL1 was also positively associated with poor outcome in primary GC (Guo et al. 2019). However, the role of ISL1 in GC proliferation and carcinogenesis, and the mechanism by which ISL1 regulates proliferation and tumorigenesis remains unclear.

In this study, we found evidence that ISL1 interacts with CDK1 in vivo and in vitro. We first confirmed ISL1 interaction with CDK1 in MGC803 cells (which showed high levels of ISL1 and proliferated more quickly) by mass spectrometry. We then determined that ISL1 interacts with CDK1/cyclin and confirmed the interaction by using a GST pull-down assay to show that ISL1 interacts directly with CDK1.

Next, we demonstrated that CDK1 specifically phosphorylates ISL1 at S269, thereby promoting the transcriptional activity of ISL1. CDK1 is a serine/threonine kinase that is often dysregulated in human cancers including GC (Gao et al. 2014; Lee et al. 2016). The ISL1 C-terminal region also contains the LIM homeobox 3 (Lhx3)-binding domain (LBD), which is a candidate intramolecular interaction domain (Gadd et al. 2013). In addition, S269 in ISL1 fits the cyclin dependent kinase 1 (CDK1) phosphorylation consensus, (S/T)PX(R/K), and is the only such motif (S269/P270) present in ISL1, although mass spectrometry analysis is required to determine the phosphorylation status of Ser269 residue in ISL1 *in vitro*. We then used the phospho-ISL1-Ser 269 antibody for immunoblot analysis of the lysates from MGC803 cells transfected with plasmids encoding CDK1, CDK1 siRNA, CDK1 (DN-CDK1), or CDK1 inhibitors. Our results indicated that Ser 269 is phosphorylated in this cell line. At the same time, we also tried to observe whether CDK1 phosphorylated ISL1 in normal human mucosal epithelial cells GES1, and it was found that the overexpression or knockdown of CDK1 greatly affected the expression of ISL1 protein (Additional file 2: Fig. S2). This may be due to the low expression of ISL1 in GES1 and the regulation of phosphorylation is different from tumor cells. We then replaced the Ser 269 residue with alanine (ISL1S269A), transfected MKN28 cells (which had low levels of ISL1 and proliferated more slowly) with wild-type or S269A-mutated ISL1. CDK1 increased phosphorylation of the wild-type ISL1 but not of the S269A mutant. Because ISL1 functions primarily as a transcription factor and forms a complex to regulate target genes (Li et al. 2014; Li et al. 2014a, b). We determined whether the ISL1-CDK1/cyclin B1 complex binds to the promoters of the target genes *CCNB*1 and *CCNB*2. ChIP and quantitative chromatin immunoprecipitation (qChIP) assays showed that the ISL1-CDK1/cyclin B1 complex showed stronger localization on *CCNB*1 and *CCNB*2 promoters. This may result in positive feedback regulation by the ISL1-CDK1/cyclin B1/2 complex in GC (Fig. 6c), although this hypothesis remains to be investigated.

We further demonstrated that CDK1-mediated modification of ISL1 may contribute to tumorigenesis and proliferation via regulation of downstream genes in GC (Benanti 2016). Up- or downregulation of ISL1 in GC cells promoted cell cycle transition into the S/G2/M phases or cell cycle arrest in the G0/G1 phases respectively. More importantly, ISL1 regulated *CCNB*1 and *CCNB*2, and consequently influenced the cell cycle. We transfected wild-type (WT) or S269A-mutated ISL1 (S269A) into MKN28 cells along with plasmids encoding CDK1 or CDK1 inhibitor RO-3306. The WT or CDK1/WT cells showed increased transcriptional activity of ISL1. Similarly, S269A or WT/RO-3306 decreased the transcriptional activity of ISL1. Importantly, the stimulatory effect of CDK1 on ISL1 was abrogated by the S269A mutation. By using stable transfects expressing wild-type ISL1 and ISL1 S269A in MKN28 cells, we observed that wild-type ISL1 cells grew faster than did ISL1 S269A cells. Consistently, the MKN28 cell cycle profiles demonstrated that ISL1 S269A overexpression remarkably decreased the cell population in the G1 phase and increased the cell population in the S and G2/M phases. Importantly, this effect was significantly more increased by transfection with wild-type ISL1. We demonstrated that phosphorylation of Ser-269 strengthens the ability of ISL1 to activate transcription and stimulate proliferation. Thus, ISL1 activity is likely linked with the cell cycle via its regulation by cyclin-dependent kinase 1, through phosphorylation of distinct sites. These results indicate for the first time that the phosphorylation status of ISL1 is essential for sustained activation of ISL1 to promote cell proliferation via regulation of downstream genes. However, detection of the mechanism by which ISL1 is activated and targeted for degradation remain to be studied. Furthermore, the potential involvement of other molecules involved in the modification of ISL1 by CDK1 remains to be clarified.

## Conclusions

We demonstrated that ISL1 interacts with CDK1, which phosphorylates ISL1 at Ser-269. ISL1-CDK1 showed improved binding on cyclin B1 and cyclin B2 promoters, and the phosphorylation increased ISL1 transcriptional activity and self-stability. It can be speculated that sustained activation of ISL1 promotes cell proliferation and tumorigenesis via a positive feedback mechanism (Fig. 6c). These findings provide, to our knowledge, the first evidence that CDK1 phosphorylates ISL1 Ser-269, and could provide a theoretical basis for the development of novel strategies and targets for GC treatment.

## Supplementary Information

**Additional file 1: Fig. S1** RO-3306 arrests cells at the G_2_/M phase border.Cell cycle profile of proliferating MGC803 (A) or treated with RO-3306 (9µM) for 20 h (B)

**Additional file 2: Fig. S2.** GES1 cells transfected with plasmid encoding CDK1 or control vector, negative control or CDK1 si-RNA. Lysate was analyzed by immunoblotting with antibodies against the indicated proteins
