# Supplementary figures and images for: Phosphorylation of islet-1 serine 269 by CDK1 increases its transcriptional activity and promotes cell proliferation in gastric cancer
Source: Mol Med. 2021 May 7;27:47. doi: 10.1186/s10020-021-00302-6 (PMC8106192; doi:10.1186/s10020-021-00302-6)

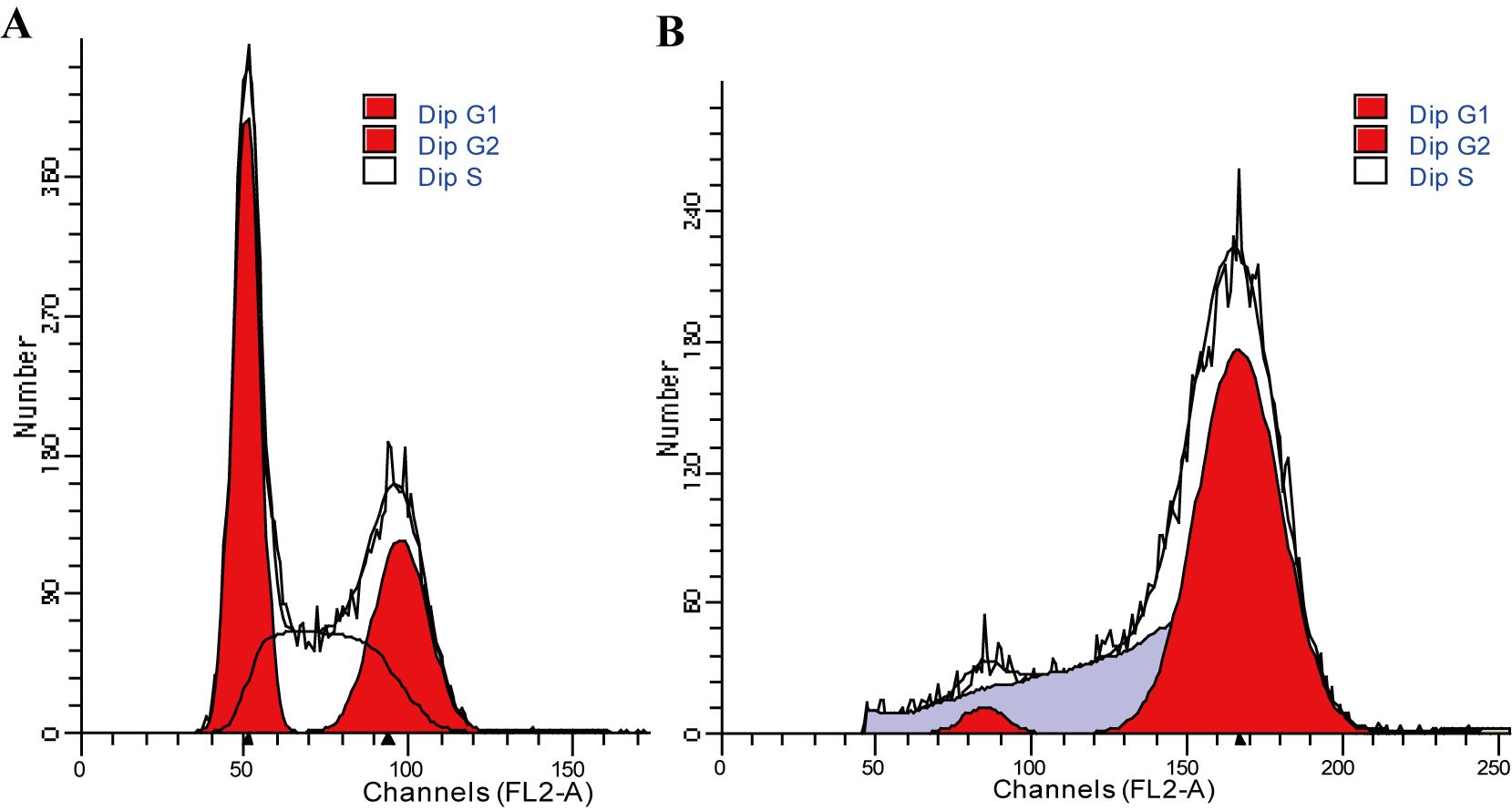

Supplement: Supplementary file 1 — Additional file 1: Fig. S1 RO-3306 arrests cells at the G2/M phase border.Cell cycle profile of proliferating MGC803 (A) or treated with RO-3306 (9µM) for 20 h (B) [file 10020_2021_302_MOESM1_ESM.tif]

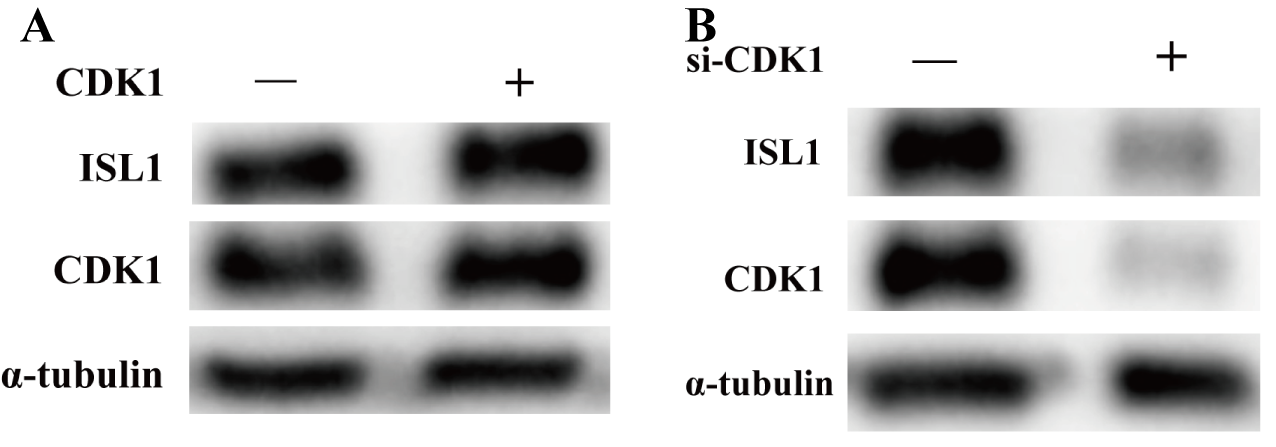

Supplement: Supplementary file 2 — Additional file 2: Fig. S2. GES1 cells transfected with plasmid encoding CDK1 or control vector, negative control or CDK1 si-RNA. Lysate was analyzed by immunoblotting with antibodies against the indicated proteins [file 10020_2021_302_MOESM2_ESM.tif]
